# High-pressure Gas Activation for Amorphous Indium-Gallium-Zinc-Oxide Thin-Film Transistors at 100 °C

**DOI:** 10.1038/srep23039

**Published:** 2016-03-14

**Authors:** Won-Gi Kim, Young Jun Tak, Byung Du Ahn, Tae Soo Jung, Kwun-Bum Chung, Hyun Jae Kim

**Affiliations:** 1 School of Electrical and Electronic Engineering, Yonsei, 50 Yonsei-ro, Seodaemun-gu, Seoul 120-.749, Republic of Korea; 2 Division of Physics and Semiconductor Science, Dongguk University, 26, Pil-dong 3-ga, Jung-gu, Seoul, 100-715, Korea

## Abstract

We investigated the use of high-pressure gases as an activation energy source for amorphous indium-gallium-zinc-oxide (a-IGZO) thin film transistors (TFTs). High-pressure annealing (HPA) in nitrogen (N_2_) and oxygen (O_2_) gases was applied to activate a-IGZO TFTs at 100 °C at pressures in the range from 0.5 to 4 MPa. Activation of the a-IGZO TFTs during HPA is attributed to the effect of the high-pressure environment, so that the activation energy is supplied from the kinetic energy of the gas molecules. We reduced the activation temperature from 300 °C to 100 °C via the use of HPA. The electrical characteristics of a-IGZO TFTs annealed in O_2_ at 2 MPa were superior to those annealed in N_2_ at 4 MPa, despite the lower pressure. For O_2_ HPA under 2 MPa at 100 °C, the field effect mobility and the threshold voltage shift under positive bias stress were improved by 9.00 to 10.58 cm^2^/V.s and 3.89 to 2.64 V, respectively. This is attributed to not only the effects of the pressurizing effect but also the metal-oxide construction effect which assists to facilitate the formation of channel layer and reduces oxygen vacancies, served as electron trap sites.

Amorphous oxide–semiconductor thin-film transistors (AOS-TFTs) have attracted much recent research attention as a substitute for amorphous silicon (a-Si) TFTs for applications as the back planes of next-generation flexible displays. AOS-TFTs exhibit a low subthreshold swing (S.S), transparency to visible light, and a high field-effect mobility (μ_FE_)^1^,^2^,^3^,^4^. In particular, amorphous indium-gallium-zinc-oxide (a-IGZO) TFTs are a promising alternative to a-Si TFTs^5^. However, with sputter-processed a-IGZO TFTs, the high energy of the target ion and incorporation of Ar^+^ ions during the sputtering process may generate scattering centers, ionized oxygen vacancies, and weak oxygen bonds, all of which deteriorate the electrical characteristics of the resulting a-IGZO TFTs^6^,^7^,^8^. Thus, an activation process using thermal energy is required to form and stabilize the channel layer of sputter-processed a-IGZO TFTs^9^. Sputter-processed a-IGZO TFTs activated at temperatures in excess of 300 °C have been commercialized as panels of liquid crystal displays and organic light-emitting diode displays since 2013. For practical applications in flexible displays, lowering the temperature of the activation process is an essential requirement during the fabrication of a-IGZO TFTs^10^,^11^. To achieve this, we employed the kinetic energy of high-pressure gases, which can supply sufficient energy to activate the a-IGZO TFTs. In previous research, we reported the use of high-pressure annealing (HPA) to improve the electrical stability of sputter-processed oxide TFTs^12^. However, to date HPA has been usually elucidated as a method to improve electrical stability in perspective of the post treatment. On the other hand, the activation mechanism of HPA for the channel layer of sputter-processed oxide TFTs has not been investigated. Hence, we intend to figure out how HPA has influence on the activation process.

In this work, we investigate the effects of HPA as an energy source to activate the a-IGZO channel layer at 100 °C in nitrogen (N_2_) and oxygen (O_2_), and also as a method to improve the electrical stability under positive bias stress (PBS). Furthermore, we describe activation mechanisms for the a-IGZO channel layer in both N_2_ and O_2_ environments through gas dynamics.

## Results

**Figure 1:**
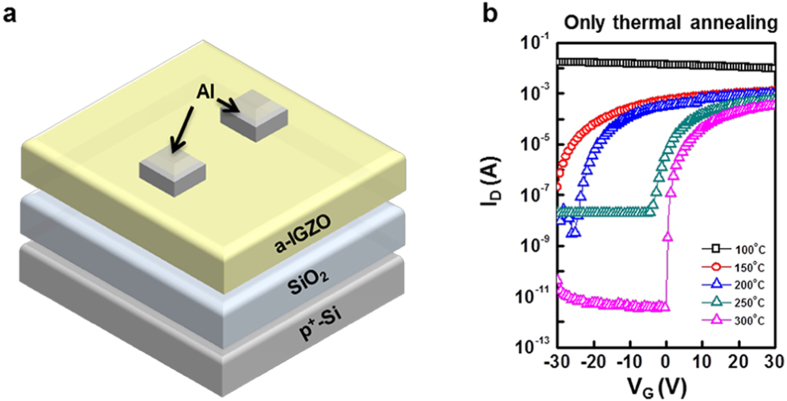
(**a**) Schematic structure of fabricated a-IGZO TFTs (**b**) Transfer characteristics of a-IGZO TFTs with only thermal annealing.

**Figure 2:**
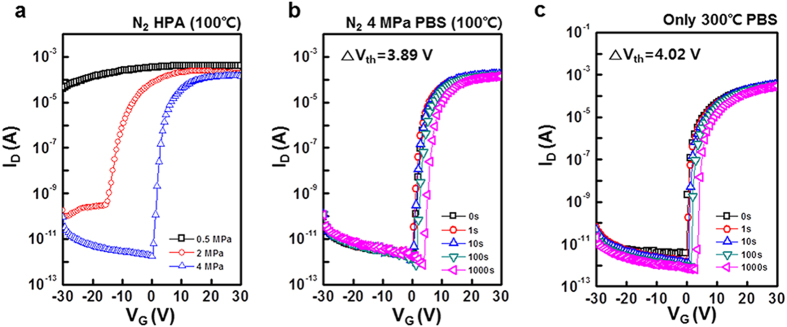
(**a**) Transfer characteristics of HPA activated a-IGZO TFTs varying N_2_ pressure at 100 °C, and PBS test results of HPA activated a-IGZO TFTs under (**b**) N_2_ 4 MPa at 100 °C (**c**) 300 °C for 1000 s.

Figure 1b shows the transfer characteristics of the thermally activated a-IGZO TFTs, which were annealed for 1 hour at 100, 150, 200, 250 and 300 °C. The TFTs annealed at ≤250 °C did not exhibit satisfactory on/off current ratios for applications as switching devices (i.e. >10^6^). Thus, the annealing temperature of sputter-processed a-IGZO TFTs should be at least 300 °C to activate the a-IGZO channel layer. The electrical characteristics of the thermally activated a-IGZO TFTs at 300 °C exhibited the following characteristics: μ_FE_ = 7.43 cm^2^/V.s, S.S = 0.39, the on/off current ratio = 1.10 × 10^8^, and the the threshold voltage (V_th_ ) = 2.08 V (Supplementary Table S1). Figure 2a shows the transfer characteristics of the HPA-activated a-IGZO TFTs annealed for 1 hour at 100 °C in N_2_ gas at pressures in the range from 0.5 to 4 MPa. The a-IGZO TFTs were activated with pressures of ≥2 MPa, and the transfer characteristics of devices activated at 4 MPa were superior to those at 2 MPa. Figure 2b,c show the results of PBS tests for the a-IGZO TFTs annealed in N_2_ at 4 MPa at 100 °C, and those annealed at 0 MPa and 300 °C. The positive V_th_ shift of the devices annealed in N_2_ at 4 MPa was 3.65 V, and that annealed at 0 MPa and 300 °C was 4.02 V. The PBS stability of the a-IGZO TFTs was also improved by annealing in N_2_. It has previously been shown that annealing in N_2_ gas improved the PBS stability of a-IGZO TFTs by reducing deep level defects in the channel layer^12^.

**Figure 3:**
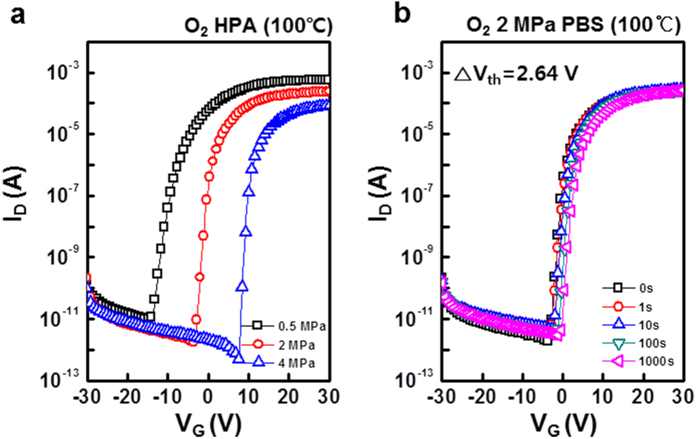
(**a**) Transfer characteristics of HPA activated a-IGZO TFTs varying O_2_ pressure at 100 °C (**b**) PBS test result of HPA activated a-IGZO TFTs under O_2_ 2 MPa at 100 °C.

These results show that HPA in N_2_ improved the PBS stability of a-IGZO TFTs, while enabling the use of a low temperature of l00 °C to form the channel. However, the required pressure of N_2_ to activate the a-IGZO channel layer was relatively high. We also investigated the use of O_2_ gas during activation, exploiting both pressure and the metal–oxide construction (MOC) effect. Figure 3a shows the transfer characteristics of HPA-activated a-IGZO TFTs annealed in O_2_ at 100 °C and pressures of 0.5, 2 and 4 MPa. The transfer characteristics of the HPA-activated a-IGZO TFTs at 2 MPa were superior to those annealed at other pressures. The positive V_th_ shift of the a-IGZO TFTs annealed in O_2_ at 2 MPa was 2.64 V, as shown in Fig. 3b. The a-IGZO TFTs annealed in O_2_ at 2 MPa exhibited superior performance compared with the devices annealed in N_2_ at 4 MPa, despite the lower pressure (Supplementary Table S1). The electrical characteristics of the TFTs annealed in O_2_ at 2 MPa were as follows: μ_FE_ = 10.58 cm^2^/V.s, S.S = 0.45, the on/off current ratio = 1.34 × 10^8^, and V_th_ = 0.48 V.

## Discussion

**Figure 4:**
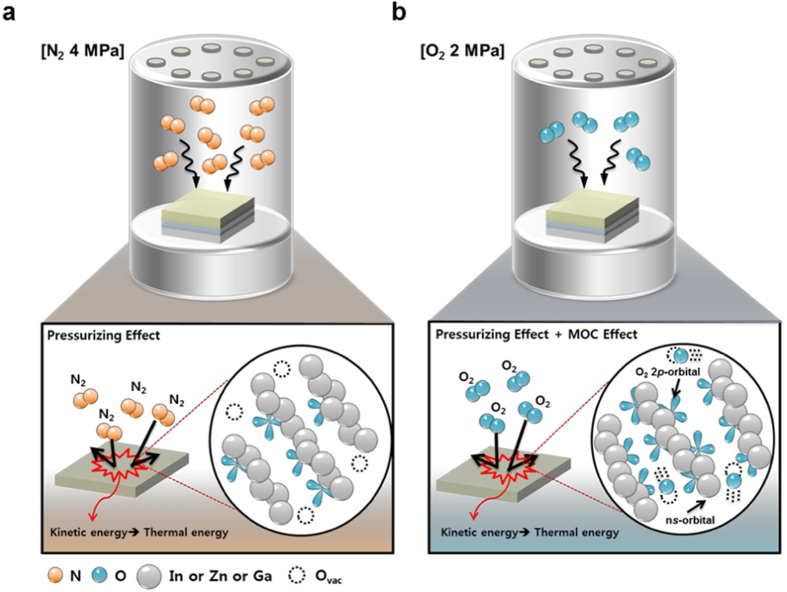
The schematic mechanism of HPA activated a-IGZO channel layer under (**a**) N_2_ 4 MPa and (**b**) O_2_ 2 MPa at 100 °C.

Figure 4a shows the activation mechanism of the a-IGZO TFTs annealed in N_2_ at 4 MPa and 100 °C, and Fig. 4b shows the activation mechanism annealed in O_2_ at 2 MPa and 100 °C. The activation of the a-IGZO channel layer in N_2_ gas can be described using the van der Waals equation, i.e.,




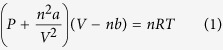




together with the ideal gas equations, i.e.,




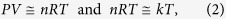




as well as the following thermodynamic relations:




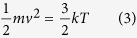




and




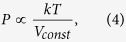




where P, V, T, k, a, b, and R are applied to gas pressure, volume of HPA chamber, temperature, Boltzmann constant, intermolecular attractive force constant, volume of molecule, and gas constant, respectively. The van der Waals equation can be used to describe the activity of a gas in the HPA chamber^13^; however, we can use the ideal gas equation because the volume of the HPA chamber is much larger than *nb* (i.e., *nb* < *V*)^14^. The average kinetic energy of gas molecules can be described using equation (3). Pressure is proportional to temperature from equations (2) and (4)^ ^15; thus, the kinetic energy of the gas molecules is equivalent to their thermal energy, and may serve as an energy source during thermal activation of the a-IGZO TFTs. We term this the “pressurizing effect”; when the pressurizing effect is applied with N_2_ gas, the a-IGZO channel layer can be activated by converting kinetic energy of the gas molecules to activation energy. The higher the pressure of N_2_ gas is applied, the greater the activation energy is supplied to activate a-IGZO channel layer. Therefore, the transfer characteristics of the HPA-activated TFTs annealed in N_2_ at 4 MPa were superior to those annealed at lower temperatures. We may expect that the collision frequency with N_2_ at 4 MPa will be double that in O_2_ at 2 MPa because it is proportional to the number of molecules^16^. Because the ratio of the molecular mass of N_2_ to O_2_ gas is 7:8, the average speed of the N_2_ molecules will be larger than that of the O_2_ gas molecules. Nonetheless, the O_2_ pressure required to activate the a-IGZO channel was half that with N_2_. This is attributed to the MOC effect as well as the pressurizing effect. The MOC effect indicates that the presence of the O_2_ gas during HPA aids the formation of direct overlapping n*s* orbitals among neighboring metals. Compared with HPA in N_2_ gas (without the MOC effect), more metal–oxygen bonds can be formed in an O_2_ environment. In addition, we could figure out that O_2_ gas improved PBS stability and had little effect on the on-current level of a-IGZO TFTs, as shown in Fig. 3b. It means that oxygen vacancies in deep levels are dominantly influenced by O_2_ gas^15^,^17^. Thus, we may expect that the density of oxygen vacancies (which act as electron trap sites) could be reduced by annealing in O_2_ gas.

**Figure 5:**
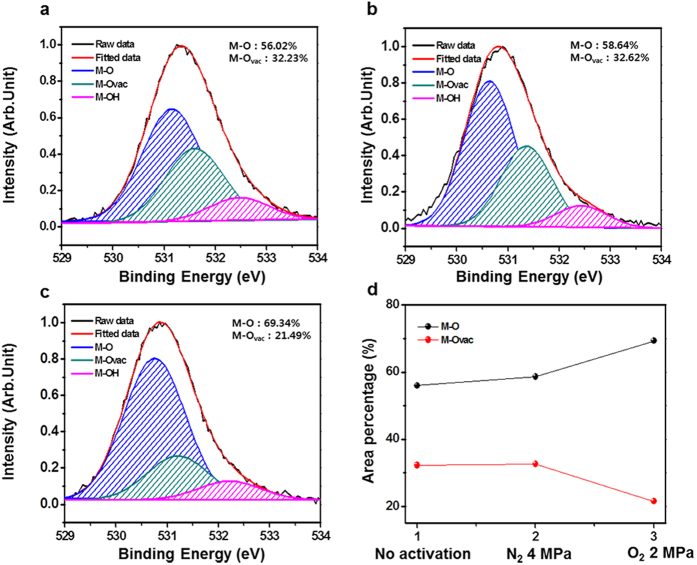
The O 1 s peaks of XPS analysis in a-IGZO channel layer under different conditions (**a**) no activation, (**b**) N_2_ 4 MPa at 100 °C, and (**c**) O_2_ 2 MPa at 100 °C. (**d**) Area ratio of M-O and M-O_vac_ in each condition.

**Figure 6:**
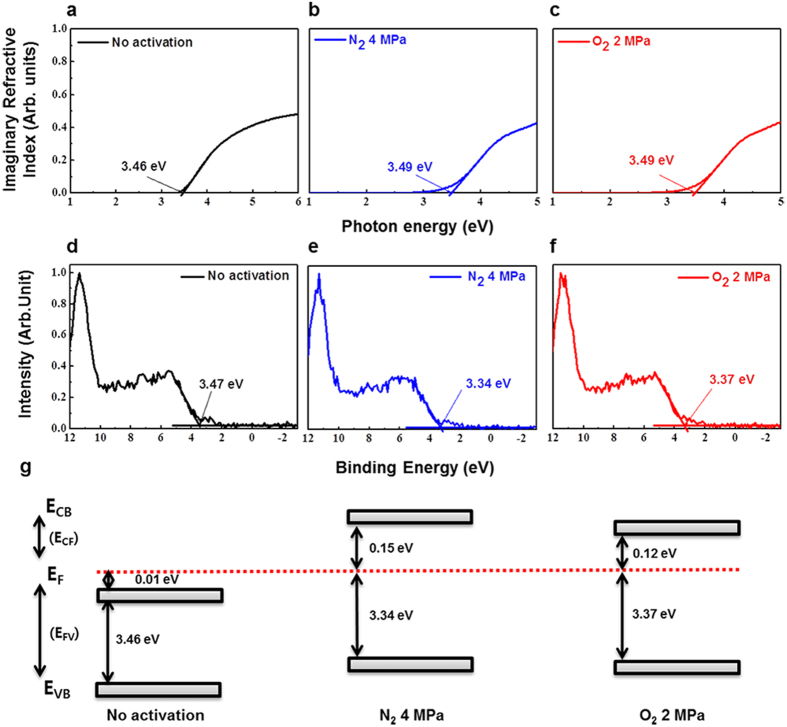
(**a–c**) Imaginary part of SE spectra absorption coefficient, (**d–f**) XPS spectra near the valence band and (**g**) band alignment of a-IGZO channel layer under different conditions: no activation, O_2_ 2 MPa at 100 °C, and N_2_ 4 MPa at 100 °C.

To investigate this mechanism of chemical composition change during HPA, we used X-ray photoelectron spectroscopy (XPS) to characterize the a-IGZO channel layers. Figure 5a–c show the O 1s peaks of the XPS spectra for the channel layers with no activation, with HPA in N_2_ at 4 MPa and 100 °C, and with HPA in O_2_ at 2 MPa and 100 °C. The ‘no activation’ indicates that a-IGZO channel layer is not influenced by thermal or pressuring process. There were three O 1 s peaks centered at 530.40 ± 0.1, 531.05 ± 0.1 and 532.35 ± 0.2 eV. These binding energy peaks indicate the metal–oxide (M-O), metal–oxygen vacancy (M-O_vac_), and metal–hydrogen lattice (M-OH) bonds, respectively. The relative area of the M-O lattice, which forms conducting pathways for charge carriers, in HPA-activated a-IGZO channel layer formed in N_2_ at 4 MPa expanded slightly compared with the non-activated channel layer (M-O increased from 56.02% to 58.64%). The ratio of M-O_vac_ lattice in the non-activated channel layer was not significantly different from that in the channel annealed in N_2_ at 4 MPa (M-O_vac_ increased from 32.23% to 32.62%), as shown in Fig. 5a,b. The N_2_ gas had little effect on the formation of the a-IGZO channel layer because of its low reactivity. It follows that N_2_ activates the a-IGZO channel layer by forming M-O bonds due to the pressurizing effect. Comparing the HPA-activated a-IGZO channel layer annealed in O_2_ at 2 MPa (see Fig. 5c) with that annealed in N_2_ at 4 MPa (see Fig. 5b), the area of the M-O lattice was significantly larger for a-IGZO channel layer annealed in O_2_ 2 MPa, whereas that of the M-O_vac_ lattice was significantly smaller (M-O increased from 58.64% to 69.34%, and M-O_vac_ decreased from 32.62% to 21.49%). We could figure out that the a-IGZO channel annealed in O_2_ at 2 MPa exhibited a higher ratio of M-O bonds compared with that annealed in N_2_ at 4 MPa. These results are summarized in Fig. 5d. It follows that more M-O bonds were formed by annealing in O_2_ at 2 MPa due to the pressurizing effect and the MOC effect. Furthermore, electron trapping is a dominant mechanism that explains the PBS stability of oxide TFTs^18^,^19^,^20^. The ionized O_vac_ defect related to M-O_vac_ leads to the PBS instabilities of oxide TFTs by forming trap sites for electrons. The a-IGZO channel layer annealed in O_2_ at 2 MPa exhibited a lower density of O_vac_ than that annealed in N_2_ at 4 MPa. This shows that HPA in O_2_ can activate the a-IGZO TFTs, as well as improve the PBS stability, using a lower pressure. Figure 6a–c show the imaginary part of the absorption coefficient from the spectroscopy ellipsometry (SE) spectra of a-IGZO channel layers with no activation, annealed in O_2_ at 2 MPa and 100 °C, and annealed in N_2_ at 4 MPa and 100 °C. The respective band gap energies were *E*_*g*_ = 3.46 eV, *E*_*g*_ = 3.49 eV and *E*_*g*_ = 3.49 eV. Figure 6d–f show the band offsets for each sample, including the Fermi energy *E*_*F*_ and the valence band energy *E*_*VB*_, which were obtained from the XPS spectra in the vicinity of the valence band energy. Using these data, we calculated the valence band offsets, as well as the differences in *E*_*F*_ and the conduction band energy *E*_*CB*_. We summarized the value of *E*_*g*_, *E*_*V*B_ − *E*_*F*_ = *E*_*FV*_, and *E*_*CB*_ − *E*_*F*_* = E*_*CF*_ for each sample (Supplementary Table S2). Figure 6g shows the band alignments of the a-IGZO channels formed with the three conditions. With no activation, the a-IGZO channel layer was degenerate, as *E*_*F*_ was located above *E*_*CB*_, and hence the channel layer was not semiconducting. The energy *E*_*CF*_ of a-IGZO channel layer annealed in O_2_ at 2 MPa was lower than those annealed in N_2_ at 4 MPa. In general, the electron concentration decreases exponentially as *E*_*CF*_ increases and the Hall mobility is proportional to the carrier concentration^21^,^22^. Furthermore, μ_FE_ is proportional to the Hall mobility in oxide semiconductors^23^,^24^,^25^. From these results, we may conclude that the μ_FE_ for the HPA-activated a-IGZO TFTs annealed in O_2_ at 2 MPa was larger than that for the devices annealed in N_2_ at 4 MPa. From the XPS and SE data, we may conclude that the sputter-processed a-IGZO TFTs should be activated for use as semiconductor devices; that the sputter-processed a-IGZO TFTs could be annealed in N_2_ at 4 MPa or O_2_ at 2 MPa and 100 °C; and that the electrical characteristics and PBS stability of the HPA-activated a-IGZO TFTs annealed in O_2_ at 2 MPa were superior to those annealed in N_2_ at 4 MPa, despite the lower pressure.

We have investigated the effects of HPA as an energy source to activate the a-IGZO channel layers as well as a method to improve the PBS stability. Activated a-IGZO TFTs were formed by annealing for 1 hour at 100 °C in N_2_ at 4 MPa, and in O_2_ at 2 MPa. The a-IGZO TFTs annealed in O_2_ required lower pressure for activation, and exhibited superior electrical characteristics. Furthermore, we investigated the mechanisms of HPA activation; i.e., the pressurizing effect and the MOC effect. We fabricated a-IGZO TFTs with excellent electrical performances and PBS stability via HPA in O_2_ at 2 MPa and 100 °C. Furthermore, we suggest that this process is feasible for the low-temperature activation of AOS-TFTs based on various flexible substrates.

## Methods

The a-IGZO TFTs were fabricated with inverted staggered structure. A SiO_2_ (1200 Å) layer was thermally oxidized on heavily boron-doped silicon (p^+^-Si) as a gate insulator. Then a-IGZO layer (40 nm) was deposited on the cleaned SiO_2_/p^+^-Si using radio-frequency (RF) magnetron sputtering at room temperature. The composition ratio of a-IGZO target was In_2_O_3_:Ga_2_O_3_:ZnO = 1:1:1. The RF power, operation pressure, and oxygen partial pressure ([O_2_]/[Ar + O_2_]) of sputtering process were fixed to 150 W, 5.0 × 10^−3^ Torr, and 0%, respectively. After a-IGZO channel was deposited on SiO_2_, the HPA was performed in different conditions: 100 °C under N_2_ (0.5, 2, and 4 MPa), and 100 °C under O_2_ gases (0.5, 2, and 4 MPa). The channel width and length were set to 1000 and 150 μm, respectively. Finally aluminum source/drain electrodes (200 nm) via shadow mask were deposited by thermal evaporation. Figure 1a shows the schematic structure of inverted staggered a-IGZO TFTs. Electrical characteristics of a-IGZO TFTs were measured by a HP 4156 C semiconductor parameter analyzer. To analyze the positive bias stability, PBS was conducted under V_GS_ = 20 V and V_DS_ = 10.1 V for 1000 s. The chemical bonding composition of a-IGZO channel layer was measured using X-ray photoelectron spectroscopy (XPS). Furthermore, we used XPS spectra near the valence band and spectroscopy ellipsometry (SE) to determine the valence band offset and the band gap energy of a-IGZO channel layer, respectively.

## Additional Information

**How to cite this article**: Kim, W.-G. *et al.* High-pressure Gas Activation for Amorphous Indium-Gallium-Zinc-Oxide Thin-Film Transistors at 100 °C. *Sci. Rep.* **6**, 23039; doi: 10.1038/srep23039 (2016).

## Supplementary Material

Supplementary Information
