# Supplementary material for: High-pressure Gas Activation for Amorphous Indium-Gallium-Zinc-Oxide Thin-Film Transistors at 100 °C
Source: Sci Rep. 2016 Mar 14;6:23039. doi: 10.1038/srep23039 (PMC4789782; doi:10.1038/srep23039)
Supplement: Supplementary Information [file srep23039-s1.pdf]

Supplementary Information

High-pressure Gas Activation for Amorphous Indium-Gallium-Zinc-Oxide Thin-Film Transistors at 100ºC

*Won-Gi Kim[1], Young Jun Tak[1], Byung Du Ahn[1], Tae Soo Jung[1], Kwun-Bum Chung[2], Hyun Jae Kim[1,]**

[1]School of Electrical and Electronic Engineering, Yonsei University, 50 Yonsei-ro, Seodaemun-gu, Seoul 120-.749, Republic of Korea

[2]Division of Physics and Semiconductor Science, Dongguk University, 26, Pil-dong 3-ga, Jung-gu, Seoul, 100-715, Korea

**hjk3@yonsei.ac.kr*

Supplementary Table S1*. Electrical characteristics of HPA activated a-IGZO TFTs under only 300 °C, O_2 2 MPa and N_2 4 MPa at 100°C.*

	μ_{FET}(cm^2/V·s)	V_{th}(V)	On/off	S.S(V/dec)	N_{it}
**No pressure + 100°C**	-	-	-	-	-
**No pressure + 300°C**	**7.43**	**2.08**	**1.10x10^8**	**2.08**	**9.48x10^{11}**
**O_2 2 MPa + 100°C**	**10.58**	**0.48**	**1.34x10^8**	**0.45**	**1.12x10^{12}**
**N_2 4 MPa + 100°C**	**9.00**	**2.97**	**8.61x10^7**	**0.50**	**1.25x10^{12}**

Supplementary Table S2*. The summarized values of E_g, △(E_{VB}-E_F), and △(E_{CB}-E_F) of a-IGZO TFTs with no activation, O_2 2 MPa, and N_2 4 MPa at 100°C.*

	E_g	△(E_{VB} – E_F)	△(E_{CB} – E_F)
**No activation**	**3.46 eV**	**3.47 eV**	**0.01 eV**
**N_2 4 MPa**	**3.49 eV**	**3.34 eV**	**0.15 eV**
**O_2 2 MPa**	**3.49 eV**	**3.37 eV**	**0.12 eV**
